# Indications for adjuvant chemotherapy in patients with AJCC stage IIa T3N0M0 and T1N2M0 gastric cancer—an east and west multicenter study

**DOI:** 10.1186/s12876-019-1096-8

**Published:** 2019-12-02

**Authors:** Ze-Ning Huang, Jacopo Desiderio, Qi-Yue Chen, Chao-Hui Zheng, Ping Li, Jian-Wei Xie, Jia-Bin Wang, Jian-Xian Lin, Jun Lu, Long-Long Cao, Mi Lin, Ru-Hong Tu, Ju-Li Lin, Hua-Long Zheng, Chang-Ming Huang

**Affiliations:** 1 Department of Gastric Surgery, Fujian Medical University Union Hospital, No. 29 Xinquan Road, Fuzhou, 350001 Fujian Province China; 2 Department of General Surgery, Fujian Medical University Union Hospital, Fuzhou, China; 3 Key Laboratory of Ministry of Education of Gastrointestinal Cancer, Fujian Medical University, Fuzhou, China; 4 Department of Digestive Surgery, St. Mary’s Hospital, University of Perugia, Terni, Italy

**Keywords:** Gastric cancer, Adjuvant chemotherapy, T3N0M0 and T1N2M0, Decision curve

## Abstract

**Purpose:**

To determine the indications for adjuvant chemotherapy (AC) in patients with stage IIa gastric cancer (T3N0M0 and T1N2M0) according to the 7th American Joint Committee on Cancer (AJCC).

**Methods:**

A total of 1593 patients with T3N0M0 or T1N2M0 stage gastric cancer were identified from the Surveillance, Epidemiology, and End Results (SEER) database for the period 1988.1–2012.12. Cox multiple regression, nomogram and decision curve analyses were performed. External validation was performed using databases of the Fujian Medical University Union Hospital (FJUUH) (*n* = 241) and Italy IMIGASTRIC center (*n* = 45).

**Results:**

Cox multiple regression analysis showed that the risk factors that affected OS in patients receiving AC were age > 65 years old, T1N2M0, LN dissection number ≤ 15, tumor size > 20 mm, and nonadenocarcinoma. A nomogram was constructed to predict 5-year OS, and the patients were divided into those predicted to receive a high benefit (points ≤ 188) or a low benefit from AC (points > 188) according to a recursive partitioning analysis. OS was significantly higher for the high-benefit patients in the SEER database and the FJUUH dataset than in the non-AC patients (Log-rank < 0.05), and there was no significant difference in OS between the low-benefit patients and non-AC patients in any of the three centers (Log-rank = 0.154, 0.470, and 0.434, respectively). The decision curve indicated that the best clinical effect can be obtained when the threshold probability is 0–92%.

**Conclusion:**

Regarding the controversy over whether T3N0M0 and T1N2M0 gastric cancer patients should be treated with AC, this study presents a predictive model that provides concise and accurate indications. These data show that high-benefit patients should receive AC.

## Background

In 2010, the American Joint Committee on Cancer/International Union Against Cancer (AJCC/UICC) published the 7th edition of the gastric cancer staging system [1]. This revision introduced a number of changes to the classification of gastric cancer, and therefore has potential clinical impact. Specifically, tumors confined to the musclaris propria (T2a) and subserosa (T2b) in the 6th edition were reclassified as T2 and T3, respectively, in the 7th edition, whereas tumors classified as pN1 in the 6th edition (1–6 involving regional lymph nodes (LNs)) were| divided into pN1 (1–2 LNs) and pN2 (3–6 LNs) in the 7th edition. Therefore, T2bN0M0 and part of T1N1M0 (stage Ib) in the 6th edition were changed to T3N0M0 and T1N2M0 (stage IIa), respectively, in the 7th edition. In most western countries, adjuvant chemotherapy (AC) is suggested in gastric cancer patients whose stage is higher than Ib [2, 3], but in eastern countries, AC is not recommended in T3N0M0 and T1N2M0 (IIa stage) gastric cancer patients [4]. Hence, the changes in the 7th edition that led to this subset of patients receiving AC are disputed [5]. Some scholars believe that stage IIa gastric cancer patients who possess certain pathological characteristics have a higher recurrence rate [6–8], but whether AC can improve the overall survival (OS) rate in this subset of patients has not been confirmed by global data. Therefore, in this article, we used the Surveillance, Epidemiology, and End Results (SEER) database to establish a forecasting model based on verified pathological features of gastric cancer patients with T3N0M0 or T1N2M0 tumors to determine who are suitable for AC. We used both east- and the west-based multicenter data with the aim of building a simple and accurate set of indications for AC in this subset of patients.

## Methods

### Study population and evaluation parameters

All analyzed data were collected from patients with gastric cancer in pathological stages T3N0M0 and T1N2M0 according to the 7th AJCC edition of the gastric cancer staging system whose data had been entered into the SEER database between January 1988 and December 2012 (Registration Number: 14088-Nov2015) or who had been seen at the Fujian Medical University Union Hospital Gastric Department (FMUUH) between October 2008 and December 2014 or the Italy IMIGASTRIC Center between January 2000 and December 2014. The following inclusion criteria were used: (1) The biopsy was confirmed as gastric cancer, (2) The only primary site was in the stomach, (3) The patient underwent radical gastrectomy, (4) The patient was treated with AC, and (5) The tumor pathological stage was T3N0M0 or T1N2M0. The following elimination criteria were applied: (1) The patient underwent radiotherapy (*n* = 10,783); (2) basic information, including race, gender, or age, was incomplete (*n* = 1989); (3) distant metastasis had developed (*n* = 21,922); (4) the pathologic diagnosis was incomplete such that stage could not be assessed (*n* = 15,122); and (5) the survival information was not clear (*n* = 4339). Finally, 1593 cases in the SEER database were included, and these were divided into an AC group (Group C) (*n* = 287) and a non-AC group (Group N) (*n* = 1306). Additionally, 241 cases from the FJUUH (198 with AC and 43 non-AC) and 45 from the IMIGASTRIC Center (22 with AC and 23 non-AC) were included.

Sociodemographic and clinicopathological data were routinely collected. The patients were divided into two groups according to age (≤ 65 and > 65 years old) based on international age standard survival classification categories [9]. The following optimal cut-off points were used to classify patients according to tumor size (the longest diameter) using the “X-tile” program: < 20 mm, ≥ 20 mm. Tumor sites were divided into three subsites, as follows: Upper third (cardiac and fundus), Middle third, and Distal third (antrum and pylorus). The tumors were pathologically categorized into well-differentiated, moderately differentiated, poorly differentiated and undifferentiated. The histological types were categorized into intestinal types and other types. Variables not mentioned in the SEER database were not included in the study and included complications, postoperative complications and incision-related complications. The pathology types were divided into adenocarcinoma and non-adenocarcinoma. OS was calculated from the date of surgery until the time of death or a follow-up termination event; when neither had yet occurred, OS was defined as deleted.

### Statistical analysis

Measurement data were analyzed using the chi-square test or Fisher’s exact probability method, and enumeration data were analyzed with the T test or Mann-Whitney U test. Survival curves were analyzed using Kaplan-Meier curves, and the Log-Rank test was used to determine differences between groups. X-tile was used to calculate the cut-off points for pathological factors and where OS was the most different between patients over and under the cut-off point. Independent risk factors that affected OS in patients with AC were determined in a Cox regression model. A nomogram for predicting OS was established. Recursive partitioning was used to determine the optimal cut-off points for the nomogram-predicted 5-year OS values. Recursive partitioning was used to objectively divide patients at each step into two groups based on predicted 5-year OS. This provided maximum survival discrimination and yielded subgroups with relatively homogeneous survival performance [10, 11]. Statistical significance was set as *P* < 0·050. All statistical analyses were performed using SPSS® Statistics for Windows® version 19.0 (IBM, Armonk, New York, USA), X-tile and R version 3.2.3 (http://www.r-project.org).

## Results

### Comparisons of overall patient characteristics between groups

Table 1 shows the comparison of characteristics between Group C and Group N in the SEER database. The results showed that the groups were significantly different according to Gender, Age, LN dissection, Size, and Histology (*P* < 0.05). The two groups did not significantly differ in AJCC staging, intestinal type, primary site, gastrectomy type or tumor grade (*P* > 0.05). Additional file 1: Table S1 shows the characteristics of patients with AC and non-AC in the FJUUH dataset. The two groups of patients exhibited significant differences in Age (*P* = 0.003) but not Gender, body mass index (BMI), American Society of Anesthesiologists (ASA) score, AJCC Operation time, Bleeding loss, LN dissection number, Size, or Primary site (*P* > 0.05). Additional file 1: Table S2 shows the comparison of characteristics of patients with AC and non-AC in the IMIGASTRIC center dataset. There were significant differences between the groups in ASA score, Approach, and Operation time (*P* < 0.05) but not Gender, Age, BMI, AJCC staging, Histology, Anastomosis method, Bleeding Loss, LN dissection number, Size, or Primary Site (*P* > 0.05).

**Table 1:** Demographic and Clinicopathologic Variables of the Adjuvant Chemotherapy and Non- Adjuvant Chemotherapy Cohorts in SEER datebase

Variable	Group C(*n* = 287)		Group N(*n* = 1306)		
	No. of Patients	%	No. of Patients	%	χ2test P
Sex					
Female	96	33.4	526	40.3	.033
Male	191	66.6	780	59.7	
Age y					
≤65	152	53.0	309	23.7	.000
>65	135	47.0	997	76.3	
AJCC					
T1N2	35	12.2	123	9.4	.157
T3N0	252	87.8	1183	90.6	
LNs dissection,No.					
≤15	140	51.2	270	20.7	.000
>15	147	48.8	1036	79.3	
Size,mm					
<20	42	14.6	669	51.2	.000
≥20	245	85.4	637	48.8	
Intestinal type					
No	247	86.1	1070	81.9	.102
Yes	40	13.9	236	18.1	
Primary Site					
Upper third	131	45.6	657	50.3	.359
Middle	47	16.4	194	13.9	
Lower third	109	38.0	455	34.8	
Gastrectomy Type					
Antrectomy	22	7.7	62	4.7	.118
Distal gastrectomy	108	37.6	544	41.7	
Upper gastrectomy	27	9.4	102	7.8	
Total gastrectomy	130	45.3	598	45.8	
Grade					
Well differentiated	7	2.4	69	5.3	.102
Moderately differentiated	79	27.5	496	38	
Poorly differentiated	192	66.9	717	54.9	
Undifferentiated	9	3.1	24	1.8	
Histology					
Adenocarcinoma	222	77.4	1125	86.1	.000
Non-Adenocarcinoma	65	22.6	181	13.9	
Follow-up,month					
Median	27		34		
Range	0-297		0-308		

Group C: Adjuvant Chemotherapy Cohort; Group N: non- Adjuvant Chemotherapy Cohort. *Abbreviations*: *LN* Lymph node, *No.* Number, *NOS* Not otherwise specified, *AJCC* American Joint Committee on Cancer, *Gx* Grade could not be evaluated

### Five-year OS of patients with AC in the SEER database

**Fig. 1:**
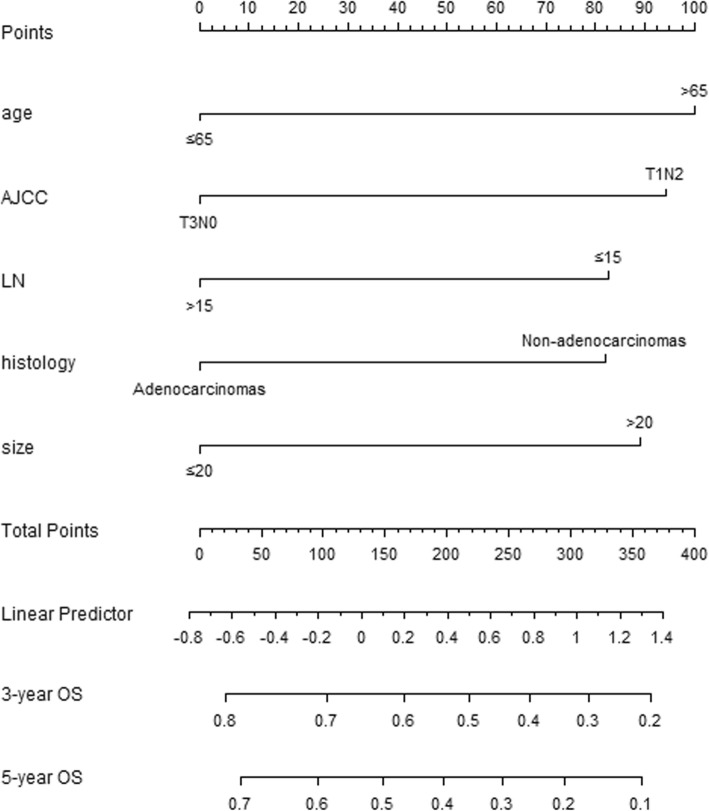
The nomogram for OS in Group C of SEER database

Additional file 1: Table S3 shows the results of univariate and multivariate Cox regression analyses, which were used to predict OS in patients with AC. After stepwise backward variable selection, only patients with Age > 65 years old, T1N2M0, LN dissection number ≤ 15, Size ≥20 mm, and nonadenocarcinoma remained in the final model (*P* < 0.05). The final model served as the basis for the multivariate nomogram (Fig. 1).

### Division of patients with AC into groups according to differences in degree of benefit

In this study, the optimal cut-off value for nomogram-predicted 5-year OS was 188 according to the recursive partitioning analysis. Patients with AC were divided into two groups, with those with points ≤ 188 regarded as high-benefit patients and those with points > 188 regarded as low-benefit patients. In addition, according to the nomogram, the low-benefit patients had some of the following features: (1) Age > 65 years old, (2) LN dissection ≤15, (3) Size ≥20 mm, (4) and nonadenocarcinoma. T1N2M0 patients with more than one of these four pathological characteristics were regarded as low-benefit patients, while T3N0M0 patients with more than three of these four pathologies were considered low-benefit patients.

### Comparison of OS between patients with different degrees of benefit and non-adjuvant chemotherapy

**Fig. 2:**
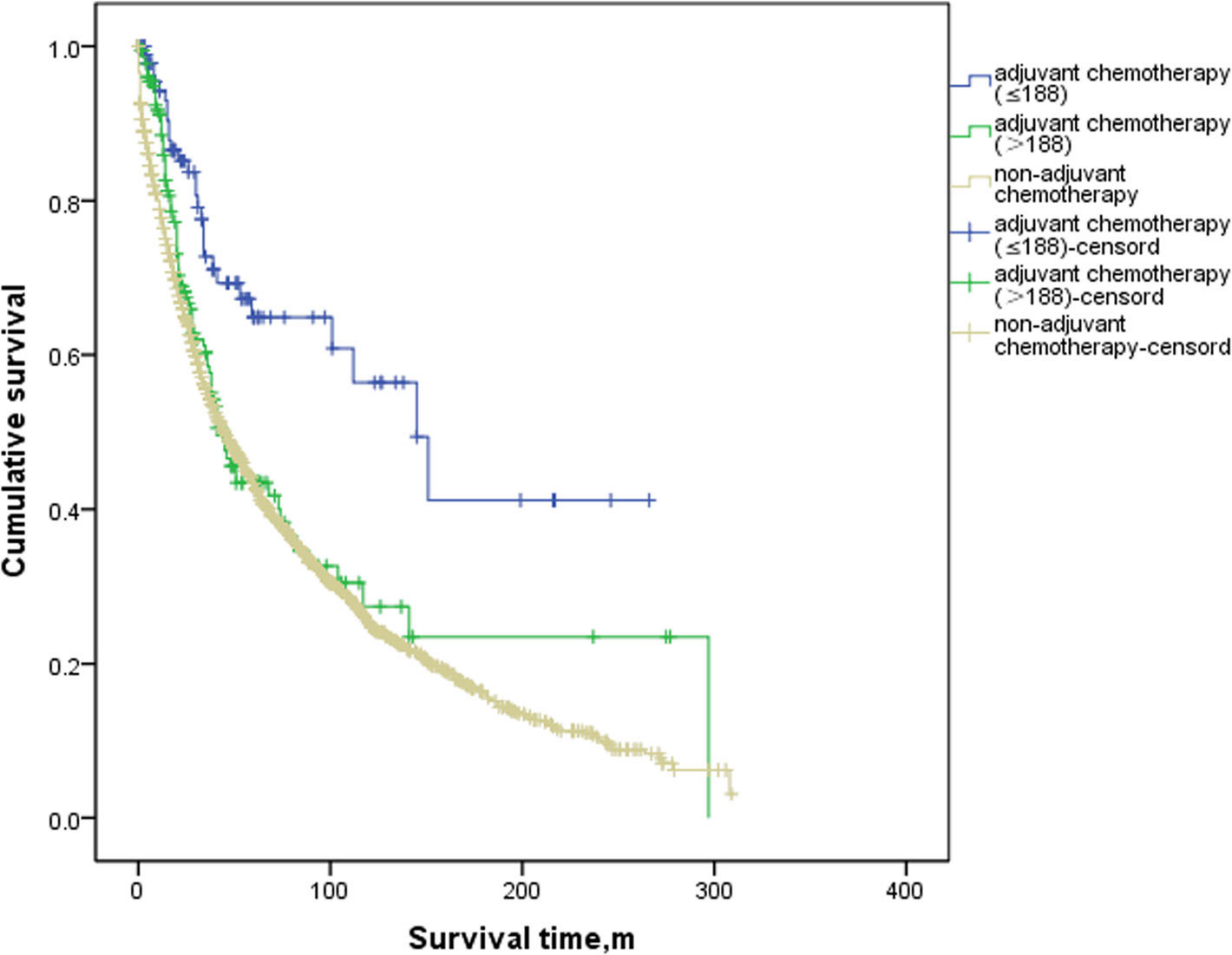
Comparisons of OS between high-benefit patients,low-benefit patients and non-Adjuvant Chemotherapy patients in SEER database. Log rank (High-benefit patients vs. low-benefit patients) =0.001. Log rank(High-benefit vs. non-Adjuvant Chemotherapy) = 0.000 and Log rank(low-benefit patients vs. non-Adjuvant Chemotherapy patients) = 0.154

**Fig. 3:**
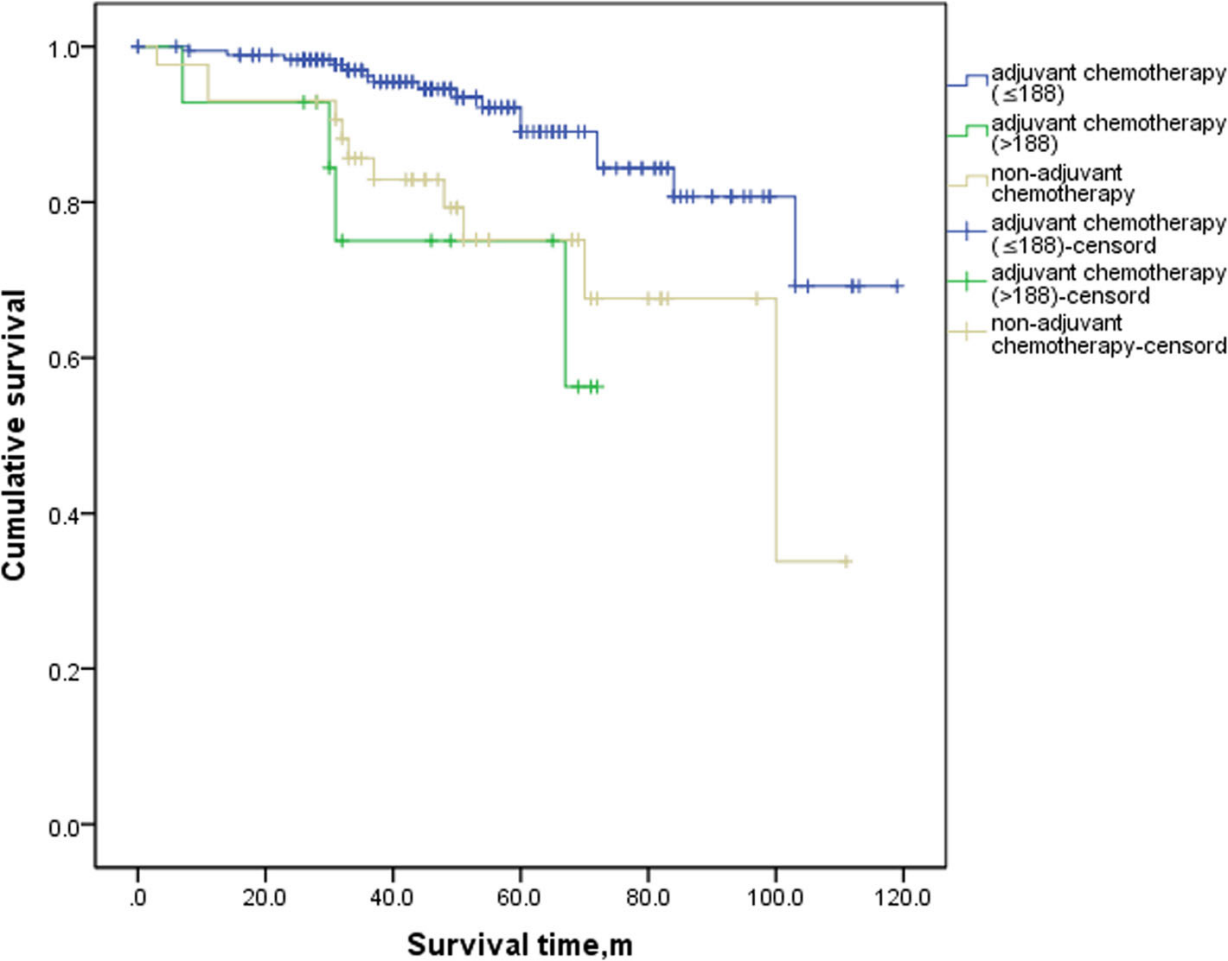
Comparisons of OS between high-benefit patients,low-benefit patients and non-Adjuvant Chemotherapy patients in FJUUH database. Log rank (High-benefit patients vs. low-benefit patients) = 0.004. Log rank(High-benefit vs. non-Adjuvant Chemotherapy) = 0.003 and Log rank(low-benefit patients vs. non-Adjuvant Chemotherapy patients) = 0.470

**Fig. 4:**
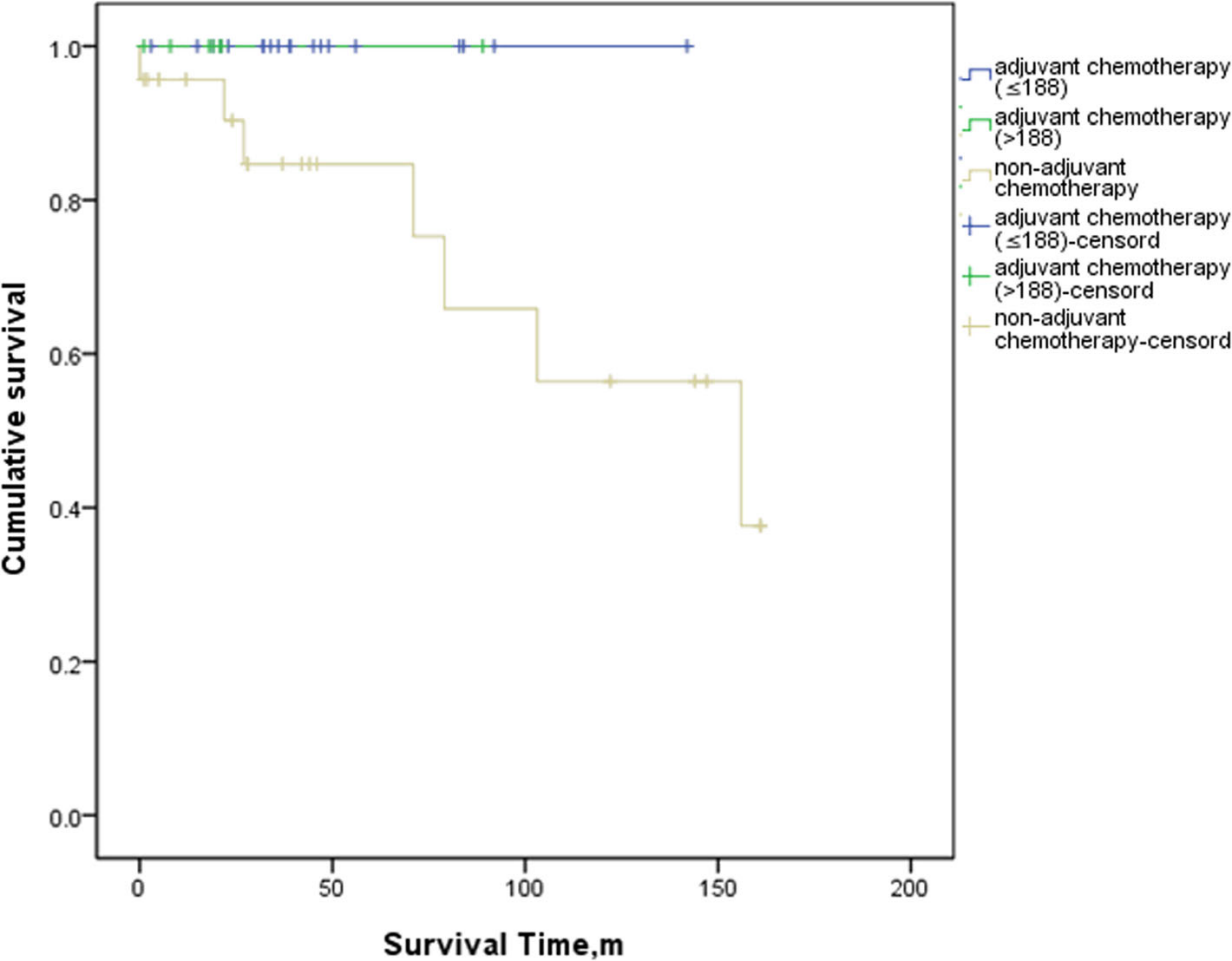
Comparisons of OS between high-benefit patients,low-benefit patients and non-Adjuvant Chemotherapy patients in IMIGASTRIC database. Log rank (High-benefit patients vs. low-benefit patients) = -. Log rank(High-benefit vs. non-Adjuvant Chemotherapy) = 0.060 and Log rank(low-benefit patients vs. non-Adjuvant Chemotherapy patients) = 0.419

In the analyses of the SEER database, the FJUUH center dataset and the IMIGASTRIC center dataset, OS was consistently better in patients with AC than in those without AC (Log-rank = 0.0001, 0.012, and 0.042, respectively) (Additional file 2: Figure S1, Additional file 3: Figure S2 and Additional file 4: Figure S3). We next compared the 5-year OS of patients in the three centers among those with a high benefit from AC, a low benefit from AC and non-AC. The results showed that in the SEER database and FJUUH datasets, OS was significantly better in the high-benefit patients than in low-benefit patients (Log-rank = 0.001 and 0.004, respectively) and the non-AC patients (Log-rank = 0.000 and 0.003) (Figs. 2 and 3). However, in the IMIGASTRIC dataset, OS was similar between the high-benefit and low-benefit patients (Log-rank = 0.060) (Fig. 4). Furthermore, there was no significant difference in OS between the low-benefit patients and non-AC patients at any of the three centers (Log-rank = 0.154, 0.470 and 0.419) (Figs. 2, 3 and 4).

### The decision curve

**Fig. 5:**
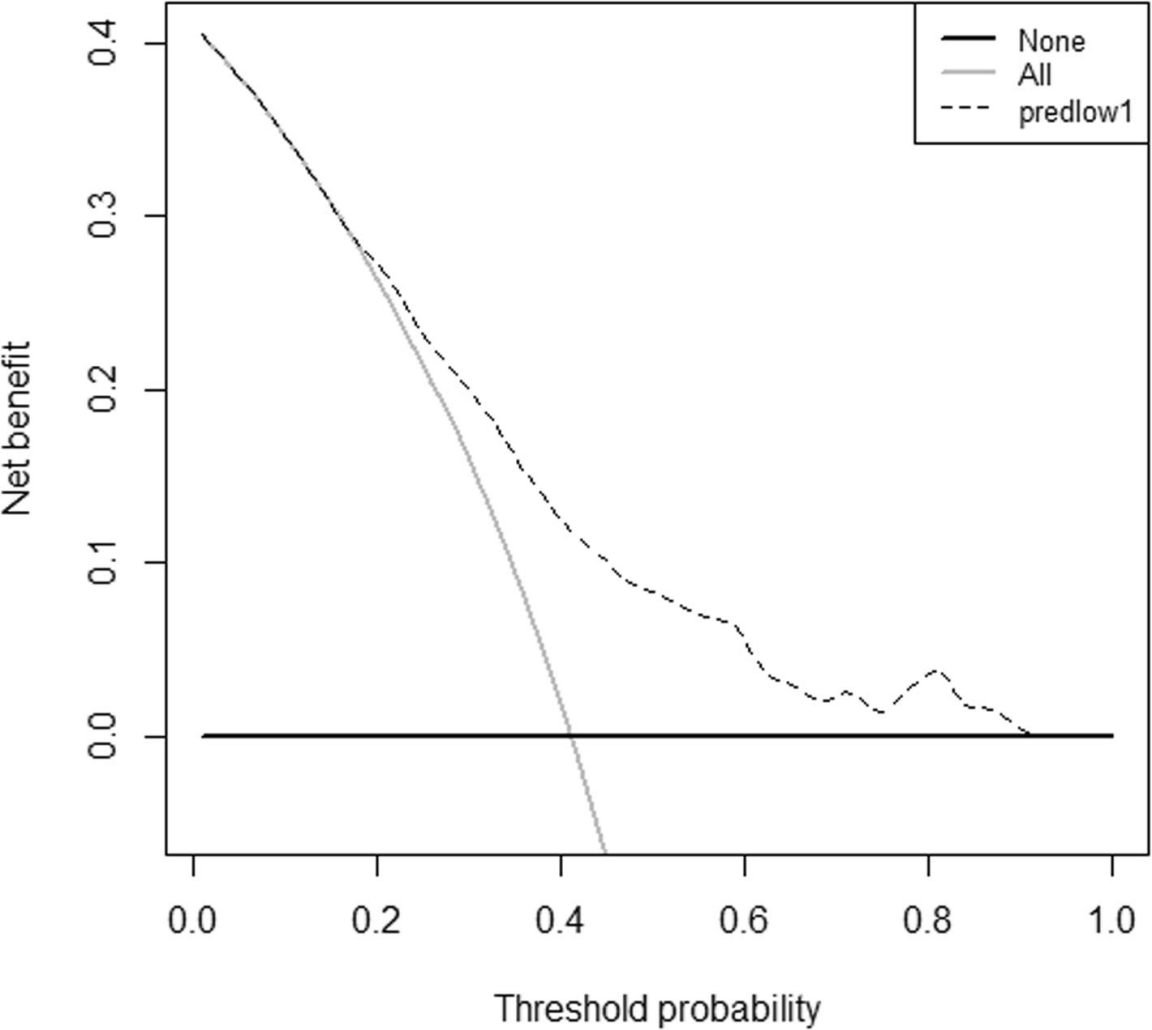
Decision curve based on the nomogram

Finally, we established a decision curve from the nomogram (Fig. 5). The results showed that the maximum benefit was obtained when the decision threshold was 0–92%, indicating that in patients with pathological stage T3N0M0 and T1N2M0, AC should be perform in patients with a score ≤ 188. Using this method, a better curative effect will be obtained than if all T3N0M0 and T1N2M0 patients do or do not undergo AC.

## Discussion

Gastric cancer is one of the most commonly malignant tumors, and the major method of curing it is surgery. The effect of D2 radical gastrectomy has been affirmed in several clinical studies performed around the world [12–14]. The findings of many important clinical research studies, including the SWOG 9008/INT-0116 Study [15], the MAGIC Study [16] and the FNLCC/FFCD Study [17], have shown that AC is beneficial for OS in gastric cancer patients. However, because the SWOG 9008/INT-0116 Study and the MAGIC study included D1 lymphadenectomy patients, while the simultaneously conducted FNLCC/FFCD Study included esophagectomy patients, doctors in eastern countries remain in doubt regarding whether the results of these clinical studies are applicable to D2 gastrectomy. The results obtained in another large clinical research study, the CLASSIC Study, showed that AC provides a benefit for OS in stage II and III gastric cancer patients after D2 gastrectomy [18]. However, the inclusion criteria of the CLASSIC Study were based on the AJCC 6th edition, ad patients with T3N0M0 and T1N2M0 according to the 7th edition could therefore not be analyzed. Therefore, whether T3N0M0 and T1N2M0 gastric cancer patients need AC and what kind of pathological characteristics indicate they will receive a benefit from AC remained to be affirmed. In this study, we used multicenter data from eastern and western datasets to explore this question. No similar report has been previously published.

In the past, the conclusions of studies [19, 20] suggesting that AC cannot increase OS in gastric cancer patients with stage IIa tumors were based simply on the effects of T and N stages. However, Waeneke [21] proposed that because TNM classification is only a mathematical model involving the simple addition of T, N and M and cannot, therefore, consider the biological characteristics of tumors, it cannot accurately reflect the actual postoperative survival of patients. Therefore, in this study, we incorporated simple and accessible tumor pathological features and discussed their effects on OS following AC with the aim of building a terse and quick-operating model that will help clinical doctors identify indications for AC in T3N0M0 and T1N2M0 gastric cancer.

Previous studies showed that a larger tumor diameter and non-adenocarcinoma increase the difficulty of R0 tumor resection, which affects postoperative OS [22, 23]. An LN dissection number less than 15 and the presence of LN metastasis increase the possibility of postoperative lymph node recurrence [24–26], which also decreases OS. These conclusions are similar to those suggested by our results. Hence, based on these factors, we built a nomogram to predict the OS of AC patients and divided the patients with AC into groups that received different degrees of benefit according to a recursive partitioning analysis. We then compared 5-year OS among patients with different degrees of benefit and non-AC patients in the SEER database, and the results showed that OS was significantly better in high-benefit patients than in low-benefit patients and those with non-AC chemotherapy. However, OS was similar between low-benefit patients and non-AC patients. The results of our analysis of the FJUUH database were the same. In the IMIGASTRIC center data, because the sample size was small, we found no significant difference between the high-benefit and low-benefit patients. We believe with a larger sample size, a significant difference would have been detected. In addition, in the IMIGASTRIC center data, OS was similar between the low-benefit and non-AC patients. These results suggest that the pathological features of high-benefit patients should be incorporated as indications for AC. However, OS was not higher in low-benefit patients who underwent AC, and the postoperative quality of life in these patients could be influenced by the toxic effects and side-effects of AC [27].

The results of this study further validate the value of using a nomogram to construct a decision curve. A decision curve is used as a simple mathematical model to use the loss of function [28] to examine the effectiveness of a statistical model for inferring the outcome of an event, and it is widely used to evaluate the usefulness and benefit of forecasting models [29–32]. The results of this study show that applying a threshold probability of 0–92% allows clinicians to achieve superior clinical effects when deciding whether a T3N0M0 or T1N2M0 patient should or should not undergo AC. A threshold probability represents the degree of confidence clinicians have in AC and the view that AC improves OS has been accepted. Hence, clinical situations should currently fall in agreement with the application scope of the nomogram, which is accord with the application scope of this decision curve. But, although the present study included a large and global sample population with long-term follow-up data, and the results obtained were further verified and validated. However, a few limitations of the study should be mentioned. First, there is inevitable bias in retrospective studies. Second, the SEER database does not include data regarding some outcomes, such as the cutting edge-positive rate and postoperative complications. Third, the number of cases and the available pathological data differed among the three centers, which may have influenced the results. Therefore, more rigorous results must been obtained in clinical trials containing multi-center, prospective and large samples.

## Conclusions

This study used multicenter data on pathological features to construct a nomogram for identifying indications for AC in patients with AJCC stage IIa gastric cancer. We believe that the nomogram established in this study can be effectively applied in clinical decision-making.

## Supplementary information

**Additional file 1: Table S1.** Demographic and Clinicopathologic Variables of Adjuvant Chemotherapy and Non- Adjuvant Chemotherapy Cohorts in FJUUH. **Table S2.** Demographic and Clinicopathologic Variables of Adjuvant Chemotherapy and Non- Adjuvant Chemotherapy Cohorts in Italy IMIGASTRIC Center. **Table S3.** Univariate and Multivariate Cox Regression Model for Prediction of Overall Survival in SEER Adjuvant Chemotherapy Cohort. Univariate and Multivariate Cox Regression Model for Prediction of Overall Survival in SEER Adjuvant Chemotherapy Cohort.

**Additional file 2: Figure S1.** Comparisons of OS between Adjuvant Chemotherapy patients and non-Adjuvant Chemotherapy patients in SEER database.Log rank = 0.0001.

**Additional file 3: Figure S2.** Comparisons of OS between Adjuvant Chemotherapy patients and non-Adjuvant Chemotherapy patients in FJUUH database.Log rank = 0.012.

**Additional file 4: Figure S3.** Comparisons of OS between Adjuvant Chemotherapy patients and non-Adjuvant Chemotherapy patients in IMIGASTRIC database.Log rank = 0.042.

## Data Availability

The datasets used and/or analyzed during the current study are available from the corresponding author on reasonable request.

## Abbreviations

AC: Adjuvant chemotherapy
AJCC: American Joint Committee on Cancer
ASA: American Society of Anesthesiologists
BMI: Body mass index
LN: Lymph nodes
OS: Overall survival
SEER: Surveillance, Epidemiology, and End Results
