# Supplementary material for: Indications for adjuvant chemotherapy in patients with AJCC stage IIa T3N0M0 and T1N2M0 gastric cancer—an east and west multicenter study
Source: BMC Gastroenterol. 2019 Dec 2;19:205. doi: 10.1186/s12876-019-1096-8 (PMC6889451; doi:10.1186/s12876-019-1096-8)
Supplement: Supplementary file 1 — Additional file 1: Table S1. Demographic and Clinicopathologic Variables of Adjuvant Chemotherapy and Non- Adjuvant Chemotherapy Cohorts in FJUUH. Table S2. Demographic and Clinicopathologic Variables of Adjuvant Chemotherapy and Non- Adjuvant Chemotherapy Cohorts in Italy IMIGASTRIC Center. Table S3. Univariate and Multivariate Cox Regression Model for Prediction of Overall Survival in SEER Adjuvant Chemotherapy Cohort. Univariate and Multivariate Cox Regression Model for Prediction of Overall Survival in SEER Adjuvant Chemotherapy Cohort. [file 12876_2019_1096_MOESM1_ESM.docx]

| **Table S1.** Demographic and Clinicopathologic Variables of Adjuvant Chemotherapy and Non- Adjuvant Chemotherapy Cohorts in FJUUH | | | | | |
| --- | --- | --- | --- | --- | --- |
| **Variable** | Adjuvant Chemotherapy Cohort**（**n=137**）** | | Non-Adjuvant Chemotherapy  Cohort**（**104**）** | |  |
|  | No. of Patients | % | No. of Patients | % | χ2test P |
| **Sex** |  |  |  |  | .628 |
| Male | 111 | 81.0 | 81 | 77.9 |  |
| Female | 26 | 19.0 | 23 | 22.1 |  |
| **Age,y** |  |  |  |  | .003 |
| ≤65 | 97 | 70.8 | 54 | 51.9 |  |
| >65 | 40 | 29.2 | 50 | 48.1 |  |
| **BMI,kg/m^2^** |  |  |  |  | .340 |
| ≤25 | 21 | 15.3 | 11 | 10.6 |  |
| >25 | 116 | 84.7 | 93 | 89.4 |  |
| **ASA scores** |  |  |  |  | .281 |
| 1 | 90 | 65.7 | 62 | 59.6 |  |
| 2 | 43 | 31.4 | 34 | 32.7 |  |
| 3 | 4 | 2.9 | 7 | 6.7 |  |
| 4 | 0 | 0 | 1 | 1.0 |  |
| **AJCC stage** |  |  |  |  | .084 |
| T1N2M0 | 23 | 16.8 | 9 | 8.7 |  |
| T3N0M0 | 114 | 83.2 | 95 | 91.3 |  |
| **Histology** |  |  |  |  | .960 |
| adenocarcinoma | 111 | 81.0 | 84 | 80.8 |  |
| Non-Adenocarcinoma | 26 | 19.0 | 20 | 19.2 |  |
| **Anastomosis method** |  |  |  |  | .846 |
| Billroth-I | 46 | 33.6 | 36 | 34.6 |  |
| Billroth-II | 7 | 5.1 | 7 | 6.7 |  |
| Roux-en-Y | 84 | 61.3 | 61 | 58.7 |  |
| **Esophagus Invasion** |  |  |  |  | .081 |
| Yes | 4 | 2.9 | 9 | 8.7 |  |
| No | 133 | 97.1 | 95 | 91.3 |  |
| **Duodenum Invasion** |  |  |  |  | .432 |
| Yes | 0 | 0 | 1 | 1.0 |  |
| No | 137 | 100 | 103 | 99.0 |  |
| **Operation Time,min** |  |  |  |  | .678 |
| ≤187 | 94 | 68.6 | 68 | 65.4 |  |
| >187 | 43 | 31.4 | 36 | 34.6 |  |
| **Bleeding Loss,ml** |  |  |  |  | .094 |
| ≤90 | 100 | 73.0 | 65 | 62.5 |  |
| >90 | 37 | 27.0 | 39 | 37.5 |  |
| **LN dissection number** |  |  |  |  | .623 |
| ≤15 | 4 | 2.9 | 2 | 1.9 |  |
| >15 | 133 | 97.1 | 102 | 98.1 |  |
| **Size,mm** |  |  |  |  | .315 |
| ＜20 | 28 | 20.4 | 16 | 15.4 |  |
| ≥20 | 109 | 79.6 | 88 | 84.6 |  |
| **Primary Site** |  |  |  |  | .321 |
| Proximal third | 44 | 32.1 | 43 | 41.3 |  |
| Mid | 28 | 20.4 | 17 | 16.3 |  |
| Distal third | 65 | 47.4 | 44 | 42.3 |  |
| **Follow-up,month** |  |  |  |  |  |
| Median | 46 | | 53 | |  |
| Range | 11-105 | | 3-119 | |  |

| **Table S2.** Demographic and Clinicopathologic Variables of Adjuvant Chemotherapy and Non- Adjuvant Chemotherapy Cohorts in Italy IMIGASTRIC Center | | | | | |
| --- | --- | --- | --- | --- | --- |
| **Variable** | Adjuvant Chemotherapy Cohort**（**n=22**）** | | Non-Adjuvant Chemotherapy  Cohort**（**23**）** | |  |
|  | No. of Patients | % | No. of Patients | % | χ2test P |
| **Sex** |  |  |  |  | .491 |
| Male | 16 | 27.3 | 19 | 82.6 |  |
| Female | 6 | 72.7 | 4 | 17.4 |  |
| **Age,y** |  |  |  |  | .763 |
| ≤65 | 9 | 40.9 | 8 | 34.8 |  |
| >65 | 13 | 59.1 | 15 | 65.2 |  |
| **BMI,kg/m^2^** |  |  |  |  | .353 |
| ≤25 | 16 | 72.7 | 13 | 56.5 |  |
| >25 | 6 | 27.3 | 10 | 43.5 |  |
| **ASA scores** |  |  |  |  | .044 |
| 1 | 6 | 27.3 | 7 | 30.4 |  |
| 2 | 13 | 59.1 | 6 | 26.1 |  |
| 3 | 3 | 13.6 | 10 | 43.5 |  |
| **AJCC stage** |  |  |  |  | .187 |
| T1N2M0 | 4 | 18.2 | 1 | 4.3 |  |
| T3N0M0 | 18 | 81.8 | 22 | 95.7 |  |
| **Histology** |  |  |  |  | .489 |
| adenocarcinoma | 22 | 100 | 21 | 91.3 |  |
| Non-Adenocarcinoma | 0 | 0 | 2 | 8.7 |  |
| **Anastomosis method** |  |  |  |  | .260 |
| Billroth II gastrojejunostomy | 6 | 27.3 | 4 | 27.3 |  |
| Jeiunal interposition | 0 | 0 | 3 | 13.0 |  |
| Roux-en-Y esophagojejunostomy | 7 | 31.8 | 10 | 43.5 |  |
| Roux-en-Y gastrojejunostomy | 9 | 40.9 | 6 | 26.1 |  |
| **Approach** |  |  |  |  | .007 |
| Open | 8 | 36.4 | 18 | 78.3 |  |
| Laparoscopic | 1 | 4.5 | 1 | 4.3 |  |
| Robotic | 13 | 59.1 | 4 | 17.4 |  |
| **Operation Time,min** |  |  |  |  | .023 |
| ≤187 | 3 | 13.6 | 11 | 47.8 |  |
| >187 | 19 | 86.4 | 12 | 52.2 |  |
| **Bleeding Loss,ml** |  |  |  |  | 1.000 |
| ≤90 | 0 | 0 | 1 | 4.3 |  |
| >90 | 22 | 100 | 22 | 95.7 |  |
| **LN dissection number** |  |  |  |  | .489 |
| ≤15 | 0 | 0 | 2 | 8.7 |  |
| >15 | 22 | 100 | 21 | 91.3 |  |
| **Size,mm** |  |  |  |  | 1.000 |
| ＜20 | 4 | 18.2 | 4 | 17.4 |  |
| ≥20 | 18 | 81.8 | 19 | 82.6 |  |
| **Primary Site** |  |  |  |  | .495 |
| Proximal third | 4 | 18.2 | 8 | 34.8 |  |
| Mid | 6 | 27.3 | 4 | 17.4 |  |
| Distal third | 12 | 54.5 | 11 | 47.8 |  |
| **Follow-up,month** |  |  |  |  |  |
| Median | 36 | | 42 | |  |
| Range | 1-142 | | 0-161 | |  |

| **Table S3**. Univariate and Multivariate Cox Regression Model for Prediction of Overall Survival in SEER Adjuvant Chemotherapy Cohort | | | | | | | | | | |
| --- | --- | --- | --- | --- | --- | --- | --- | --- | --- | --- |
|  | **Univariate Model** | | | | | **Full Multivariate Model** | | | | |
| **Variable** | **OR** | **95%CI** | | | **P** | **OR** | **95%CI** | | | **P** |
| **Sex** |  |  |  |  |  |  |  |  |  |  |
| Female | Ref |  |  |  |  | Ref |  |  |  |  |
| Male | .948 | .645 | - | 1.394 | .788 | .983 | .657 | - | 1.473 | .935 |
| **Age y** |  |  |  |  |  |  |  |  |  |  |
| ≤65 | Ref |  |  |  |  | Ref |  |  |  |  |
| >65 | 1.695 | 1.170 | - | 2.454 | .005 | 1.820 | 1.243 | - | 2.666 | .002 |
| **AJCC** |  |  |  |  |  |  |  |  |  |  |
| T1N2 | Ref |  |  |  |  | Ref |  |  |  |  |
| T3N0 | 0.560 | .359 | - | .871 | .010 | .556 | .353 | - | .874 | .011 |
| **LNs dissection,No.**  ≤15  ＞15 |  |  |  |  |  |  |  |  |  |  |
|  | Ref  0.602 |  | | | .008 | Ref  0.660 | .443 |  |  |  |
|  |  | .414 | - | .875 |  |  |  | - | .983 | .041 |
| **Size mm** |  |  |  |  |  |  |  |  |  |  |
| ＞20 | Ref |  |  |  |  | Ref |  |  |  |  |
| ≤20 | .527 | .325 | - | .855 | .009 | .596 | .352 | - | .977 | .040 |
| **Intestinal type** |  |  |  |  |  |  |  |  |  | .107 |
| No | Ref |  |  |  |  | Ref |  |  |  |  |
| Yes | .644 | .336 | - | 1.234 | .185 | .728 | .434 | - | 1.792 | .882 |
| **Primary Site** |  |  |  |  |  |  |  |  |  |  |
| Upper third | Ref |  |  |  |  | Ref |  |  |  |  |
| Middle | .887 | .484 | - | 1.627 | .699 | 1.065 | .570 | - | 1.991 | .844 |
| Lower third | 1.180 | .802 | - | 1.736 | .401 | 1.453 | .961 | - | 2.197 | .077 |
| **Grade** |  |  |  |  |  |  |  |  |  |  |
| Well differentiated | Ref |  |  |  |  | Ref |  |  |  |  |
| Moderately differentiated | 7369 | .000 | - | - | .884 | 7201 | .000 | - | - | .888 |
| Poorly differentiated | 8965 | .000 |  | - | .882 | 8387 | .000 |  | - | .886 |
| Undifferentiated | 9241 | .000 | - | - | .881 | 9932 | .000 | - | - | .884 |
| **Histology adenocarcinoma** |  |  |  |  |  |  |  |  |  |  |
| Non- adenocarcinoma | Ref |  |  |  |  | Ref |  |  |  |  |
| Adenocarcinoma | .595 | .401 | - | .883 | .010 | .610 | .395 | - | .942 | .026 |
