# Supplementary figures and images for: Indications for adjuvant chemotherapy in patients with AJCC stage IIa T3N0M0 and T1N2M0 gastric cancer—an east and west multicenter study
Source: BMC Gastroenterol. 2019 Dec 2;19:205. doi: 10.1186/s12876-019-1096-8 (PMC6889451; doi:10.1186/s12876-019-1096-8)

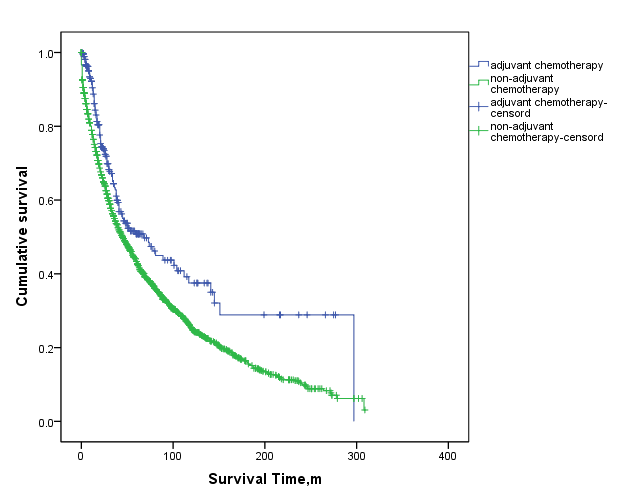

Supplement: Supplementary file 2 — Additional file 2: Figure S1. Comparisons of OS between Adjuvant Chemotherapy patients and non-Adjuvant Chemotherapy patients in SEER database.Log rank = 0.0001. [file 12876_2019_1096_MOESM2_ESM.tif]

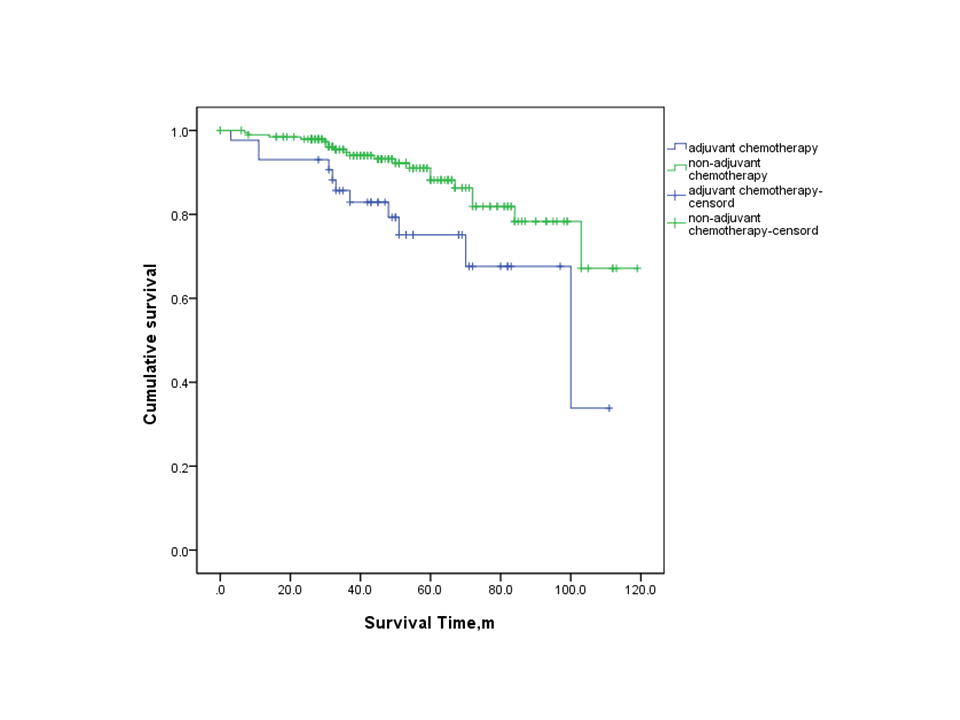

Supplement: Supplementary file 3 — Additional file 3: Figure S2. Comparisons of OS between Adjuvant Chemotherapy patients and non-Adjuvant Chemotherapy patients in FJUUH database.Log rank = 0.012. [file 12876_2019_1096_MOESM3_ESM.tif]

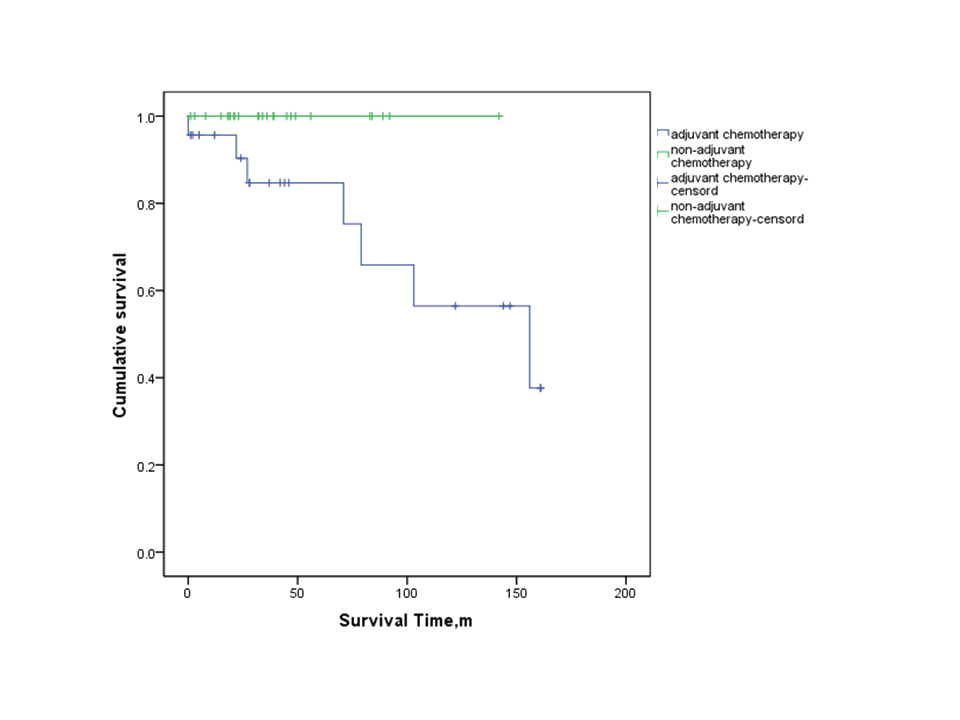

Supplement: Supplementary file 4 — Additional file 4: Figure S3. Comparisons of OS between Adjuvant Chemotherapy patients and non-Adjuvant Chemotherapy patients in IMIGASTRIC database.Log rank = 0.042. [file 12876_2019_1096_MOESM4_ESM.tif]
